# The effect of D-serine administration on cognition and mood in older adults

**DOI:** 10.18632/oncotarget.7691

**Published:** 2016-02-24

**Authors:** Marcos Avellar, Linda Scoriels, Caroline Madeira, Charles Vargas-Lopes, Priscila Marques, Camila Dantas, Alex C. Manhães, Homero Leite, Rogerio Panizzutti

**Affiliations:** ^1^ Biomedical Sciences Institute, Federal University of Rio de Janeiro, RJ, Brazil; ^2^ Institute of Psychiatry, Federal University of Rio de Janeiro, RJ, Brazil; ^3^ Department of Psychiatry, University of Cambridge, Cambridge, United Kingdom; ^4^ Institute of Biology Roberto Alcantara Gomes, Biomedical Center, State University of Rio de Janeiro, RJ, Brazil; ^5^ Integrated Unit for Prevention, Adventist Silvestre Hospital, Rio de Janeiro, RJ, Brazil

**Keywords:** D-serine, cognition, aging, glutamate metabolism, mood, Gerotarget

## Abstract

**Background:**

D-serine is an endogenous co-agonist of the N-Methyl D-Aspartate Receptor (NMDAR) that plays a crucial role in cognition including learning processes and memory. Decreased D-serine levels have been associated with age-related decline in mechanisms of learning and memory in animal studies. Here, we asked whether D-serine administration in older adults improves cognition.

**Results:**

D-serine administration improved performance in the Groton Maze learning test of spatial memory and learning and problem solving (F_(3, 38)_= 4.74, *p* = 0.03). Subjects that achieved higher increases in plasma D-serine levels after administration improved more in test performance (r^2^=−0.19 *p* = 0.009). D-serine administration was not associated with any significant changes in the other cognitive tests or in the mood of older adults (*p* > 0.05).

**Methods:**

Fifty healthy older adults received D-serine and placebo in a randomized, double blind, placebo-controlled, crossover design study. We studied the effect of D-serine administration on the performance of cognitive tests and an analogue mood scale. We also collected blood samples to measure D-serine, L-serine, glutamate and glutamine levels.

**Conclusions:**

D-serine administration may be a strategy to improve spatial memory, learning and problem solving in healthy older adults. Future studies should evaluate the impact of long-term D-serine administration on cognition in older adults.

## INTRODUCTION

Normal aging is generally accompanied by a decline in several domains of cognitive function, which are significantly associated with functional limitations [1-3]. Specifically, impairments in executive functions are a major contributor to the functional limitation associated with aging [4-7]. Executive functions include the ability of reasoning, planning and executing goal-directed behaviors, and the maintenance of these functions is a critical target to promote healthy aging.

The frontal cortex mediates executive function impairments, and the neural circuits that are vulnerable to aging are composed primarily of glutamatergic neurons [8]. This system plays a crucial role in cognitive functions *via* several receptors including the N-methyl-D-aspartate (NMDA) receptor [9]. NMDA receptor activity depends on both the binding of glutamate and the activation of a co-agonist site by glycine or D-serine [10].

Evidence indicates that D-serine is associated with age-related cognitive decline. Animal studies have demonstrated a marked decrease of D-serine levels in the hippocampus of aged rats, resulting in reduced NMDA receptor activity [11-13]. This decrease in D-serine contributes to age-related deficits in cellular mechanisms that are related to memory and learning in rodents [14]. Importantly, our group demonstrated that plasma D-serine levels decrease with aging in a cohort of healthy subjects spanning from 19 to 72 years of age [15]. The association between age-related decline in brain functions and reductions in D-serine levels suggest that D-serine administration in older adults may improve brain functions that are affected by aging.

This study aimed to investigate the effects of a single D-serine dose in healthy older adults in a randomized, double blind, placebo-controlled, crossover-designed study. First, we tested the effect of D-serine administration on functions that are affected by aging and depended on proper NMDA receptor function such as learning and memory [16, 17], problem solving [18-20], and working memory [21]. Second, we investigated the effect of D-serine administration on visual attention, which is frequently impaired in the older adult population and can affect performance on the other computerized tests. Third, we assessed the subjective mood state during testing sessions. This assessment was exploratory and primarily used to account for any change in participant mood during the cognitive tests. The effect of D-serine administration in emotion processing is relatively unknown, and it was only recently reported that D-serine administration reduced anxiety and sadness in healthy young adults [22].

## RESULTS

### Demographics

Participants were predominantly women (37 women and 13 men), who were 73 years old with an IQ of 110 and 12 years of education on average. D/P and P/D groups had similar demographics, and the groups did not differ statistically in any of these variables (Table 1).

**Table 1:** Demographics and neuropsychological description of the study sample

	All participants *n* = 50	P/D group* n = 25	D/P group† *n* = 25	*p*-value‡
Age (mean (SD))	73 (5)	74 (5.2)	72 (4.7)	0.24
Gender (female, male)	37, 13	17, 7	20, 6	0.69
Education (mean (SD))	12 (4)	13 (4.2)	12 (3.8)	0.32
WAIS IQ (mean (SD))	110 (11)	111 (10)	110 (12)	0.92
GDS Yesavage (mean(SD))	1.62 (1.54)	1.44 (0.30)	1.8 (0.32)	0.42
MMSE (mean (SD))	29 (1.12)	29.1 (1.09)	28.8 (1.15)	0.29

* P/D group: participants who received placebo on the first session and D-serine on the second session.

† D/P group: participants who received D-serine on the first session and placebo on the second session.

‡ p-value represents the statistical result of the difference tested between P/D and DP groups.

GDS: Geriatric Depression Scale; MMSE: Mini Mental State Examination; SD: Standard Deviation, WAIS IQ: Weschler Adult Intelligence Scale Intelligence Quotient.

### Effect of D-serine administration on executive function, working memory, attention and mood

**Figure 1:**
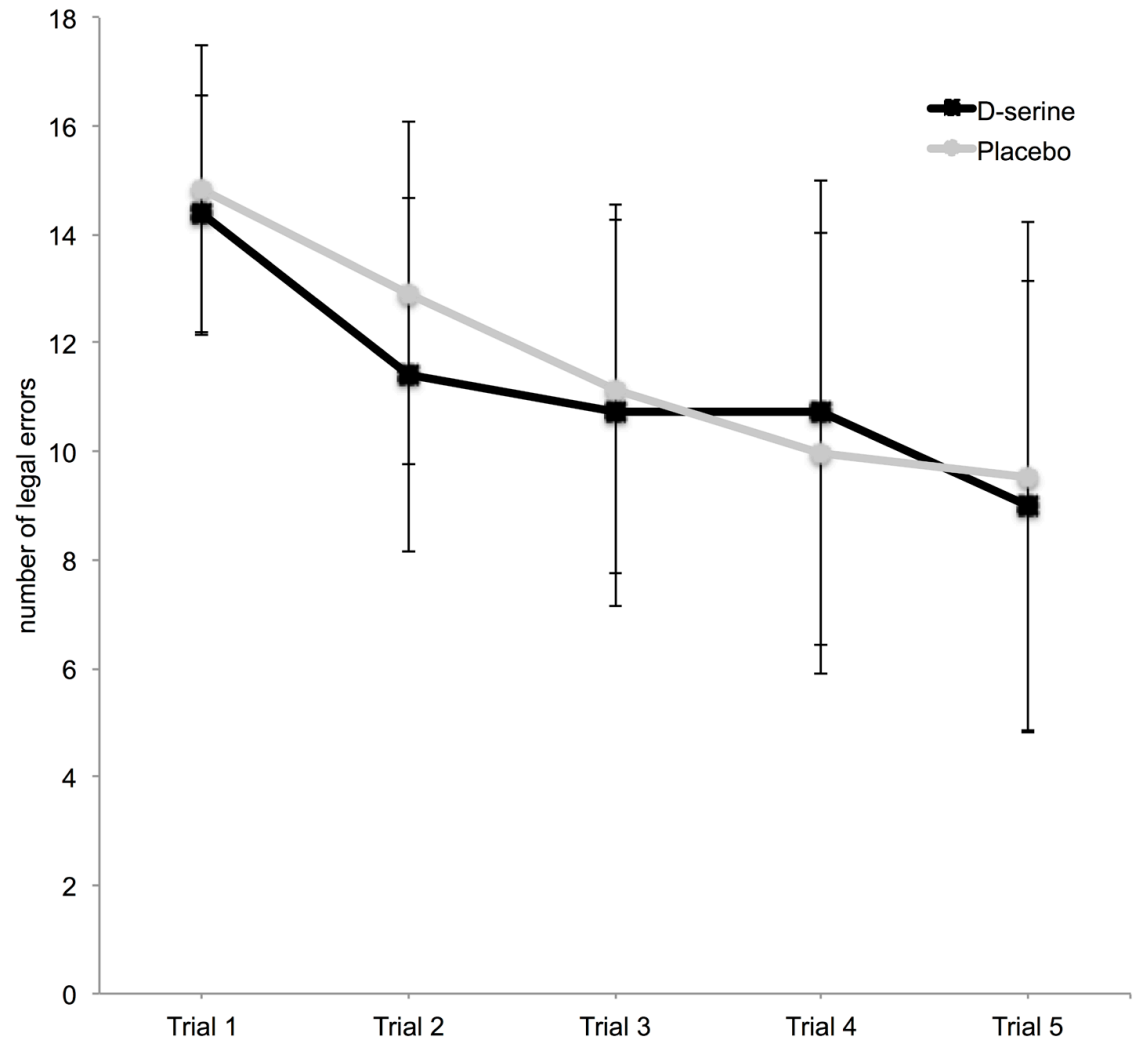
Legal errors on the Groton Maze Learning test (GML) on D-serine and placebo ** *p* < 0.01. Bars represent standard deviation.

D-serine administration was associated with a statistically significant decrease in legal errors on the Groton Maze Learning test for spatial memory, learning and problem solving (F_(3, 38)_ = 4.74, *p* = 0.03, Figure 1). *Post hoc t*-tests showed that the second trial had the most statistically significant difference between the two groups (t = −3.13, *p* = 0.003, Figure 1). There were no significant effects of D-serine administration on illegal errors (F_(3, 38)_ = 0.05, *p* = 0.82), indicating that the effect was not due to differences in the comprehension of the test.

D-serine administration did not have any statistically significant effect on the key measures of the n-back working memory test (accuracy, *p* > 0.1), the Set-Shifting cognitive flexibility task (extra-dimensional shift errors, *p* = 0.47), or the visual attention test (errors, *p* > 0.7). D-serine administration also had no effect on the mood visual analogue scale (*p* > 0.4). D-serine-mediated effects on outcome measures related to cognition are summarized on Table 2.

**Table 2:** Effect of D-serine administration on cognitive performance

Cognitive Test / Measure	Placebo mean (SD)	D-serine mean (SD)	Effect size (d)	*p*-value
				
Groton Maze Learning Test				
Legal errors	58.38 (2.09)	56.00 (2.04)	1.15	0.03
Illegal errors	21.13 (3.18)	21.27 (2.86)	0.05	0.82
N-back				
One-back accuracy	0.90 (0.01)	0.88 (0.01)	1.27	0.12
Two-back accuracy	0.80 (0.02)	0.78 (0.02)	1.01	0.57
Set Shifting				
Extradimensional reverse errors	4.92 (0.43)	5.29 (0.48)	0.81	0.47
Visual Attention				
Errors	6.68 (1.01)	6.16 (0.67)	0.62	0.32

### Plasma levels of D-serine and other amino acids

We measured the levels of D-serine and other amino acids related to the glutamatergic system in the plasma of subjects who had received placebo. Several amino acids demonstrated a weakly (all r^2^ below 0.07) significant negative association with age. There was a marginally statistically significant negative association between D-serine levels and age (*r*^2^ = −0.04, *p* = 0.09). There was a statistically significant negative association between glutamate levels and age (*r*^2^ = −0.07, *p* = 0.04) and L-serine levels and age (r^2^ = −0.07, p = 0.04). There was no correlation between glutamine levels and age (*r*^2^ = 0.03, *p* = 0.17).

We also analyzed the plasma amino acid levels after D-serine administration. D-serine administration induced marked changes in D-serine levels (z = −5.30, *p* < 0.00001) but had no impact on other amino acids such as glutamate (z = −0.25, p = 0.80), glutamine (z = −0.01, *p* = 0.99), and L-serine (z = −0.49, *p* = 0.63).

**Figure 2:**
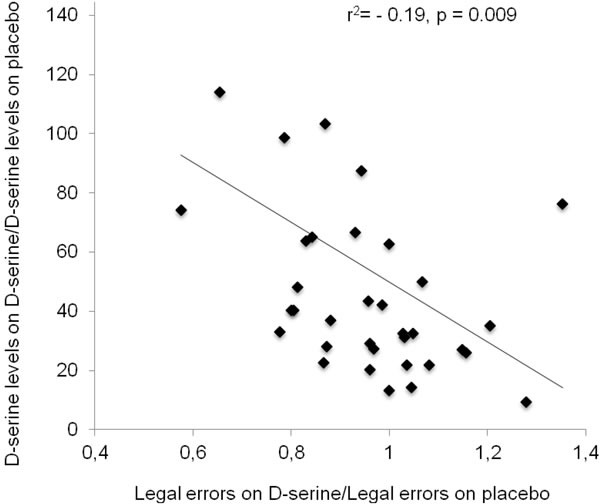
Association between the ratios of D-serine on D-serine over placebo and the number of legal errors on the Groton Maze Learning test on D-serine over placebo Pearson's correlation shows a negative correlation (*r*^2^ = −0.19, *p* = 0.009).

We analyzed the degree of association between the cognitive improvement observed in the Groton Maze Learning test and D-serine levels. *Post hoc* correlations between the ratio of D-serine levels on D-serine and placebo and the ratio of legal errors from the Groton Maze Learning test demonstrated a negative association between the two outcome measures (r^2^ = −0.19 *p* = 0.009) (Figure 2).

## DISCUSSION

The present study revealed that a single D-serine dose improved executive function, specifically spatial memory, learning and problem solving in older adults. We did not observe a significant effect of D-serine administration on verbal working memory, visual attention, cognitive flexibility and mood. Several studies investigated the effect of exogenous D-serine administration both in rodents and in humans. In adult rodents, D-serine administration improves several modalities of memory, including spatial and working memory [23-25]. In aged rodents, D-serine counterbalanced the effect of aging on several process including synaptic plasticity and memory [14]. D-serine administration improved cognition in patients with schizophrenia [26-28]. Recently, Levin and co-workers demonstrated that D-serine administration improved attention, verbal learning and memory as well as subjective feelings of sadness and anxiety in young healthy adults [22]. Our results in older adults highlight the D-serine site of the NMDA receptor as a potential therapeutic target for remediation of the age-related decline in executive functions.

The remediation of age-related cognitive decline may benefit from the association of pharmacological compounds that enhance learning and cognitive training. In our study, D-serine treatment improved performance in the earlier phase of learning in the Groton Maze Learning test, indicating that D-serine may be useful to improve learning when associated with cognitive training strategies. However, it is noteworthy that D-serine had no significant effect in a pilot study of computerized cognitive training in schizophrenia subjects [29]. The best strategies to potentially improve NMDA receptor co-activation with D-serine or other substances as well as the best cognitive training to be combined with substance administration remain to be elucidated.

We also observed an association between the ratio of the increase in D-serine levels after administration and the change in performance induced by D-serine on the Groton Maze Learning test. Thus, subjects who achieved higher increases in plasma D-serine levels after administration demonstrated larger improvements in cognition. These results suggest that its poor oral bioavailability can limit the effects of D-serine on cognition. Accordingly, one study in schizophrenia demonstrated that D-serine had better effects on cognition when administered in higher doses such as 60 and 120 mg/kg [26]. In contrast, another study failed to demonstrate an effect of D-serine on the response to cognitive training in schizophrenia with a dose of 30 mg/kg [29]. In general, poor oral D-serine bioavailability may account for mixed results in clinical trials [26-28], indicating that larger doses should be evaluated. However, the safety of D-serine administration in higher doses to older adults was not assessed.

With the exception of the Groton Maze Learning test, subjects improved their performance on the second session of testing compared with the first session regardless of when D-serine was administered, indicating the possibility of a practice effect (Figure 1 supplement). Thus, it may be that practice affects on attention, working memory and cognitive flexibility tests washed out the effect of D-serine. The Groton Maze Learning test may have overcome this problem because the test itself includes practice within its design.

Given the role of D-serine in NMDA receptor co-activation, one may expect that its reduction would impair cognition. D-serine is markedly decreased in the hippocampus of aged rodents [13]. Likewise, our group observed that plasma D-serine levels are inversely associated with aging in a population of healthy subjects spanning from 19 to 72 years old [15]. In the present study, we observed a non-significant tendency (*p* = 0.09) for an inverse association of D-serine levels and age in participants ranging from 65 to 85 years old. The absence of significance may be related to the limited span of age in the group studied. More studies are needed to define whether this age-related decrease in peripheral D-serine levels that seems to occur in humans also happens at the level of the central nervous system. Most importantly, it would be of interest to determine whether reductions of D-serine levels, aging and cognitive deficits are part of the same process.

This study has some limitations: the effect of D-serine on episodic memory and autobiographical memory, among other types of memories remains unknown. D-serine administration did not demonstrate an effect on other cognitive functions, which may be related to the dose administered. Thus, future studies in older adults should evaluate the effect of different D-serine doses on cognition. It would also be of interest to evaluate the effects of longer periods of D-serine administration and to determine whether the gains in cognitive function are maintained once the treatment is finished.

Aging with a good quality of life is a very modern challenge, and there is an ongoing debate about how to address the age-related decline in brain functions that lead to incapacity. Oral ingestion of one D-serine dose improved spatial memory, learning and problem solving in older adults. This result confirms that the age-related changes in brain function are not static, and it is indeed possible to stop or partially reverse the plastic processes that are involved in age-related cognitive decline. Improvements in cognition as observed with D-serine administration may help older adults keep their independence in daily activities, thus contributing to a better quality of life [30-32]. Indeed, different cognitive training in the areas of perceptual discrimination, working memory and fluid intelligence in older adults has demonstrated evidence of transfer-of-benefit to daily activities and quality of life [33-35]. The remediation of age-related cognitive decline may benefit from the replacement of missing elements in the machinery such as D-serine, which is associated with appropriated cognitive training.

## MATERIALS AND METHODS

### Participants and procedure

In total, fifty-seven older adult individuals were recruited from the Integrated Prevention Unit in Silvestre Adventist Hospital in Rio de Janeiro, Brazil. We posted advertisements, and subjects self-referred. Inclusion criteria were older than 65 years and clinically healthy. Exclusion criteria were use of acetylcholinesterase inhibitors, two or more falls in the previous 6 months before entering the study, history of any neuropsychiatric disorder, a Mini Mental State Examination (MMSE) score below 27, a Geriatric Depression Scale of Yesavage (GDS) score above 5, and an IQ score below 70. From the 57 subjects recruited, 50 participants completed the entire study protocol (Table 1). Of the participants, seven withdrew from the study during or after the first session: 5 had health conditions between the first and second sessions, one could not attend the second session, and one was afraid of ingesting the substance.

The study was approved by the ethics committee of the Federal University of Rio de Janeiro (411/09), and all of the participants gave written informed consent. It was a randomized, double blind, placebo-controlled, crossover-design study. Of the participants, one half were randomized to receive an oral dose of 30 mg/kg D-serine diluted in orange juice on the first session followed by an oral dose of placebo (only orange juice) in the second session (D/P group). The other half of the participants was randomized to receive placebo in the first session followed by D-serine in the second session (P/D group). The taste of D-serine in the orange juice was not noticeable. Sessions were at least one week apart, which is reportedly sufficient to minimize practice effects. A dose of 30 mg/kg D-serine has been well tolerated in various other studies with no report of significant side effects [26-28].

Volunteers were tested from May 2010 to August 2011. They completed a personal interview that asked information about their height and weight, physical activities, drug use history, handedness, and familiarity with computers. Subjects who did not know how to use a computer were trained beforehand. IQ was estimated using the Wechsler adult intelligence scale (WAIS) subtest with Vocabulary and Matrix Reasoning [36].

On the first visit, participants received D-serine or placebo, waited 1.5 hours and then performed computerized cognitive tests. On the second visit, cognitive tests were performed again using parallel versions whenever possible to avoid practice effects. On both visits, participants answered a mood visual analogue scale before administration and after the cognitive test. After testing, we collected a sample of peripheral blood.

### Computerized cognitive tests

We used computerized cognitive tests provided by Cogstate (www.cogstate.com). These included the Groton Maze Learning test, which assesses spatial memory, learning and problem solving. Working memory was assessed with the one-back and the two-back tasks. We used the Set-Shifting task to assess cognitive flexibility. We also applied a computerized visual attention test to assess sustained visual attention [37].

### Statistics

To assess the effects of D-serine relative to placebo, we used repeated-measures analysis of variance (ANOVA) with a type III full factorial model. Normality and homogeneity of data distribution were confirmed using the Kolmogorov-Smirnov and Shapiro-Wilk and Levine tests, respectively. Where appropriate transformations did not result in normal distributions, the non-parametric Wilcoxon test was used.

RM-ANOVA was performed to analyze results from the Groton Maze Learning test. Intervention (2 levels, D-serine and placebo) and blocks (5 levels, trial 1 to 5) were defined as within-subject factors, and the order of D-serine and placebo administration (P/D or D/P) was defined as a between-subject factor. Some results indicated an intervention-by-order effect, in which case, the first and second session data were analyzed separately *post hoc* using parametric (multivariate or one-way) ANOVA or non-parametric Mann-Whitney *U* tests. We also performed *post hoc* analysis of the effect of the order of D-serine and placebo administration using a paired *t*-test.

A one-way ANOVA was used to compare age and IQ between groups (P/D and D/P), and the Wilcoxon non-parametric test was used for the remaining demographics, biomarkers and cognitive data.

Correlations were performed using Pearson's r. One-tailed Pearson's correlations were used to test the association of age and biomarkers of the glutamatergic system as there is an *a priori* hypothesis that these two factors are related. Two-tailed Pearson's correlations were used for the remaining association analyses. Data were analyzed with SPSS software version 22 for Mac.

Due to operational constraints during testing and blood collection, data were not available for 1 participant on the Groton Maze learning test and 1 participant on the N-back task, and 8 blood samples could not be tested.

## SUPPLEMENTARY MATERIAL FIGURE


