# Supplementary material for: The effect of D-serine administration on cognition and mood in older adults
Source: Oncotarget. 2016 Feb 24;7(11):11881–8. doi: 10.18632/oncotarget.7691 (PMC4914255; doi:10.18632/oncotarget.7691)
Supplement: Supplementary file 1 [file oncotarget-07-11881-s001.pdf]

The effect of D-Serine administration on cognition and mood in older adults

Supplementary Material

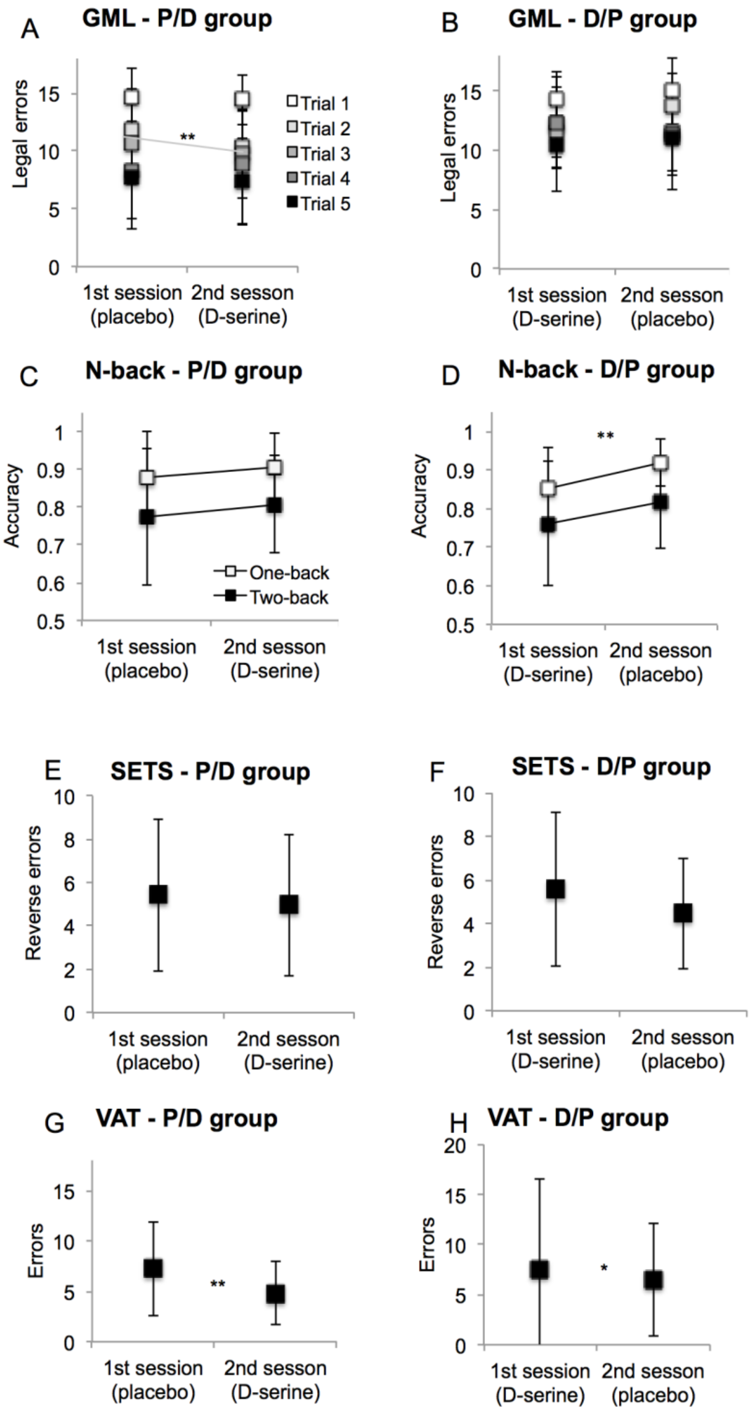

Supplement Figure 1: Cognitive performance separated by group who performed the tests under placebo on the first session and under D-serine on the second sessions (P/D) and by group who performed the tests under D-serine on the first session and under placebo on the second session (D/P). **A.** Performance on the Groton Maze Learning test (GML) for the group P/D. **B.** Performance on the GML for the group P/D. **C.** Performance on the N-back task for the group P/D. **D.** Performance on the N-back task for the group D/P. **E.** Performance on the Set Shifting Test (SETS) for the group P/D. **F.** Performance on the SETS for the group D/P. **G.** Performance on the Visual Attention Task (VAT) task for the group P/D. **H.** Performance on the VAT task for the group D/P. * $p < 0.05$, ** $p < 0.01$. Bars represent standard deviation.
